# Supplementary material for: Giant isotropic magneto-thermal conductivity of metallic spin liquid candidate Pr2Ir2O7 with quantum criticality
Source: Nat Commun. 2021 Jan 12;12:307. doi: 10.1038/s41467-020-20562-w (PMC7804409; doi:10.1038/s41467-020-20562-w)
Supplement: Supplementary file 1 — Supplementary Information [file 41467_2020_20562_MOESM1_ESM.pdf]

Supplementary materials for "Giant isotropic magneto-thermal conductivity of metallic spin liquid candidate $Pr_2Ir_2O_7$ with quantum criticality"

J. M. Ni[1], Y. Y. Huang[1], E. J. Cheng[1], Y. J. Yu[1], B. L. Pan[1], Q. Li[1], L. M. Xu[2], Z. M. Tian[2,*] and S. Y. Li[1,3,4,*]

[1] *State Key Laboratory of Surface Physics, Department of Physics, and Laboratory of Advanced Materials, Fudan University, Shanghai 200438, China*

[2] *School of Physics, and Wuhan National High Magnetic Field Center, Huazhong University of Science and Technology, Wuhan 430074, China*

[3] *Collaborative Innovation Center of Advanced Microstructures, Nanjing 210093, China*

[4] *Shanghai Research Center for Quantum Sciences, Shanghai 201315, China*

Supplementary Note 1: X-ray diffraction measurement

The typical x-ray diffraction (XRD) data is plotted in Supplementary Fig. 1, determining the largest surface of the samples to be the (111) plane. The heat current is applied in the (111) plane.

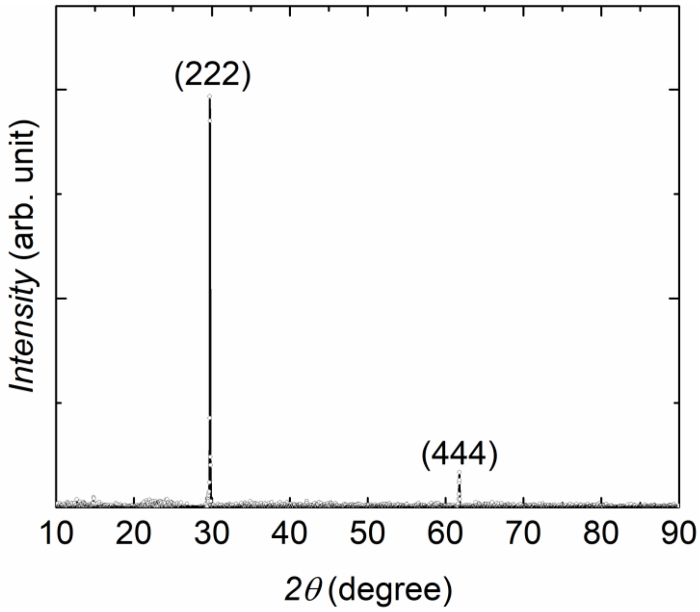

Supplementary Figure 1 | Typical x-ray diffraction pattern of the $Pr_2Ir_2O_7$ single crystal. The largest plane is determined to be the (111) plane.

Supplementary Note 2: Scaling of thermal conductivities

Interestingly, an unusual scaling behavior κ - $\kappa_e \sim H^3$ F($T/H^{4/3}$) is observed, as shown in Supplementary Fig. 2. $\kappa_e = L_0T/\rho(H)$ is the electron thermal conductivity at each field. Thus, κ - κ_e represents the thermal conductivity of phonon part, since there is no magnetic thermal conductivity in $Pr_2Ir_2O_7$, as illustrated in the main text. F(x) is the scaling function. It is also held for sample B (see Supplementary Fig. 4b). Note that the x variable $T/H^{4/3}$ in the function F(x) is exactly the same as the scaling law found in the magnetic Grüneisen ratio. Observation of the scaling law in the thermal conductivity is appealing since it is rather rare that the heat transport data scale as a function of a single parameter. It gives a new viewpoint towards such quantum magnets that we hope will stimulate the theoretical study.

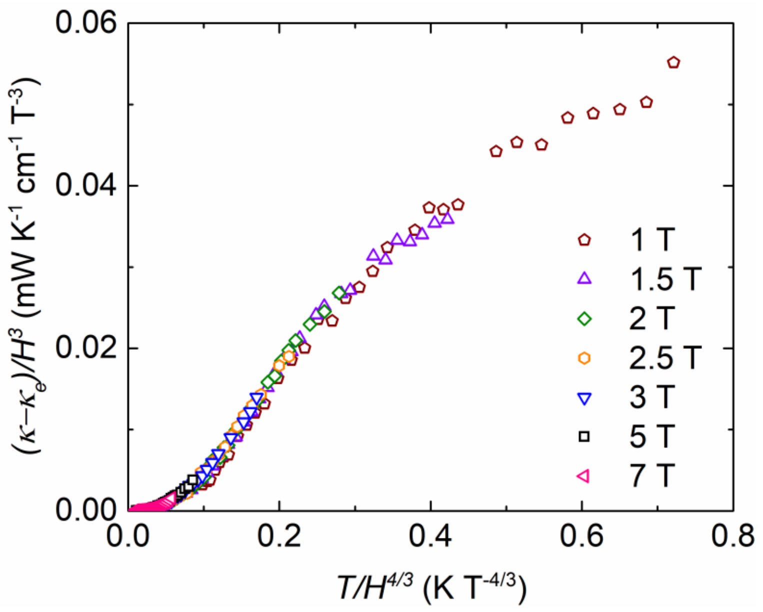

Supplementary Figure 2 | Scaling plot of the thermal conductivity of phonon part. The plots of ($\kappa-\kappa_e)/H^3$ versus $T/H^{4/3}$ at various magnetic fields parallel to the [111] direction collapse on the same curve.

Supplementary Note 3: Reproducibility of heat transport results

We performed transport measurements on another $Pr_2Ir_2O_7$ single crystal (Sample B), and obtained similar results to Sample A. Sample B was cut and polished into a rectangular shape with length l = 0.72 mm, width w = 0.26 mm and thickness t = 0.20 mm. The electric and thermal conductivity were measured by standard four-wire method.

Supplementary Fig. 3a shows the temperature dependence of the longitudinal resistivity of Sample

B at zero field. The Kondo effect is also evidenced by the minimum at 45 K (see the inset of Supplementary Fig. 3a), confirming the good quality of our sample. The plots of $\rho(T)$ at 0, 3, and 5 T are presented in Supplementary Fig. 3b. By extrapolating to the zero-temperature limit, we can get the residual resistivity ρ_0 = 757, 743, and 755 μΩ cm for $\mu_0 H$ = 0, 3, and 5 T, respectively.

Supplementary Fig. 4a shows the thermal conductivities of Sample B up to 7 T. The magnetic fields were applied along the [111] direction. Just as Sample A, the thermal conductivities overlap with each other and deviate from the high-field data above particular temperatures T_s. The same scaling law $\kappa - \kappa_e \sim H^3 \, F(T/H^{4/3})$ is also found in Sample B, see Supplementary Fig. 4b.

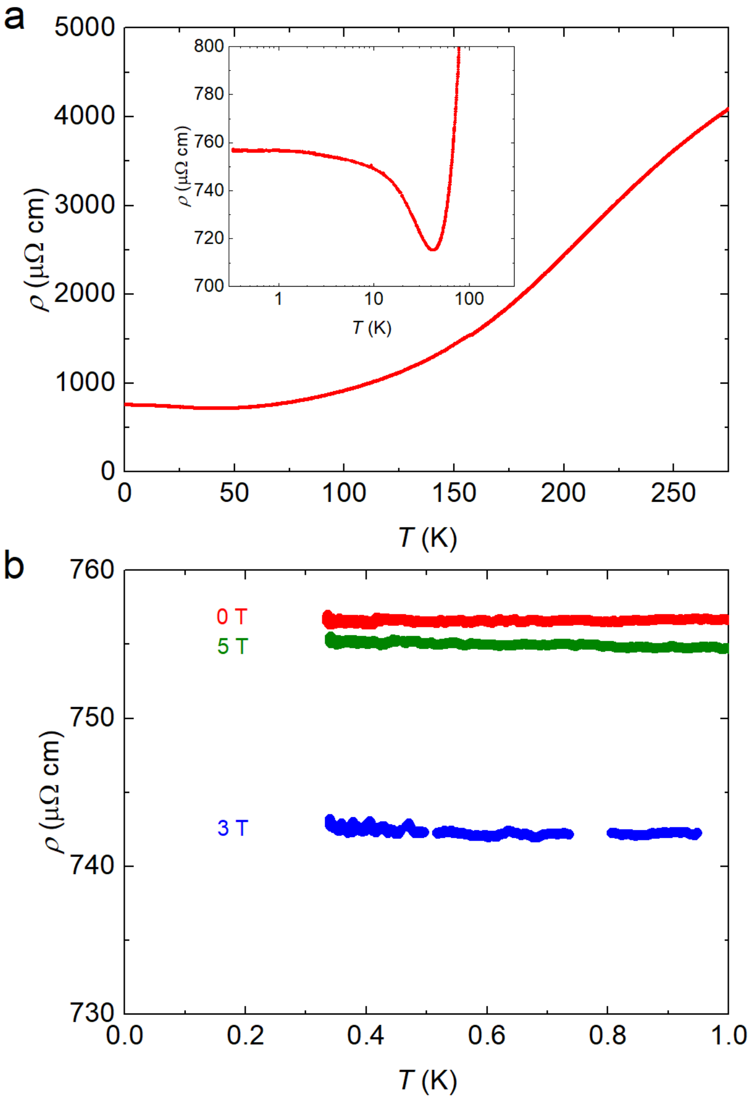

Supplementary Figure 3 | Charge transport results of Sample B. a Temperature dependence of the resistivity of $Pr_2Ir_2O_7$ Sample B. The inset: zoomed view of the resistivity minimum at 45 K due to the Kondo effect. **b** $\rho(T)$ below 1 K in $\mu_0 H$ = 0, 3, and 5 T. The magnetic fields were applied along the [111] direction.

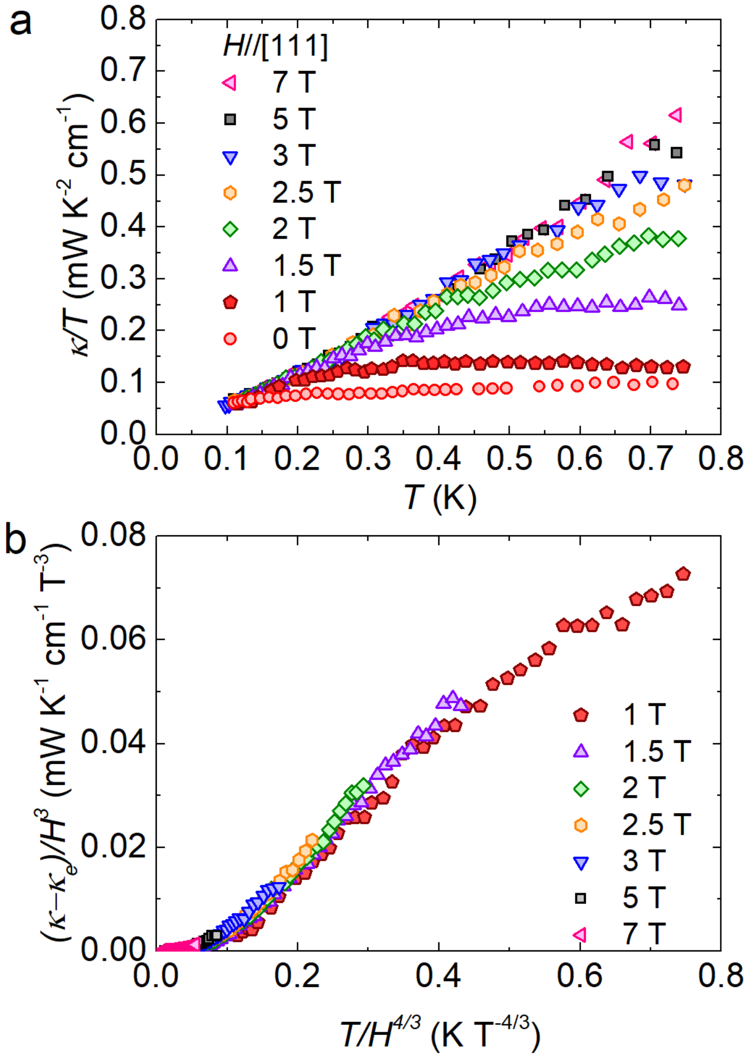

Supplementary Figure 4 | Heat transport results of Sample B. a The thermal conductivity of Sample B at various magnetic fields along the [111] direction. **b** Scaling plot of the thermal conductivity of phonon part from Sample B. The plots of $(\kappa - \kappa_e)/H^3$ versus $T/H^{4/3}$ at various magnetic fields parallel to the [111] direction collapse on the same curve.

Supplementary Note 4: High-temperature thermal conductivities

The high-temperature thermal conductivities of Sample B are shown in Supplementary Fig. 5a. The magnetic fields are applied along the [111] direction. A broad peak is observed at around 10 K at each field. The κ/T data at $\mu_0 H$ = 5 T still overlap with those at 7 T below 9 K, indicating that besides the electron thermal conductivity, the κ above 5 T is purely contributed by phonons without magnetic scatterings until thermal fluctuations of spins dominate over polarization effects by magnetic fields at even higher temperatures (T > 9 K). Unexpectedly, contrary to the ultralow-temperature results, the zero-field data are the largest among the data at other fields and the κ data are suppressed more strongly with increasing the fields. In other words, negative MTCs are observed at temperatures above 5 K, which is opposite to the giant positive MTCs at sub-Kelvin region. Therefore, there must exist a crossover from the positive MTCs at low temperatures to the negative MTCs at high temperatures between 1 K and 5 K, as shown in the yellow part of Supplementary Fig. 5b.

This crossover region is in good correspondence with the energy scale of renormalized interactions between Pr moments, which is θ_w = 1.7 K. When $T < \theta_w$, the susceptibility and Hall resistivity start to exhibit logarithmically diverging behaviors and the anomalous Hall effect emerges (Ref. 6-8 in the main text). The magnetic specific heat also exhibits a broad peak at this temperature (Ref. 6 in the main text). These all indicate a spin-liquid state below this temperature. This particular energy scale comes from the partial screening of Pr moments due to the Kondo effect, which renormalizes the AFM interaction from the RKKY interaction energy scale of about 20 K to θ_w = 1.7 K. As a result, θ_w is a critical temperature dividing two separate states: when $T < \theta_w$, the underscreened moments form a correlated spin liquid, where "2-in, 2-out" configurations are the ground states; when $T > \theta_w$, the Kondo effect starts to lead to the screening of the 4f moments (Ref. 6,8 in the main text). Therefore, the crossover at this temperature region may be due to the different physical states above and below θ_w. In this context, the origin of the negative MTC at high temperatures must be different from the positive MTC at low temperatures, and should be related to the Kondo screening of 4f moments. Note that the negative MTC itself is rather unexpected and interesting, and calls for a careful research and analysis in the future.

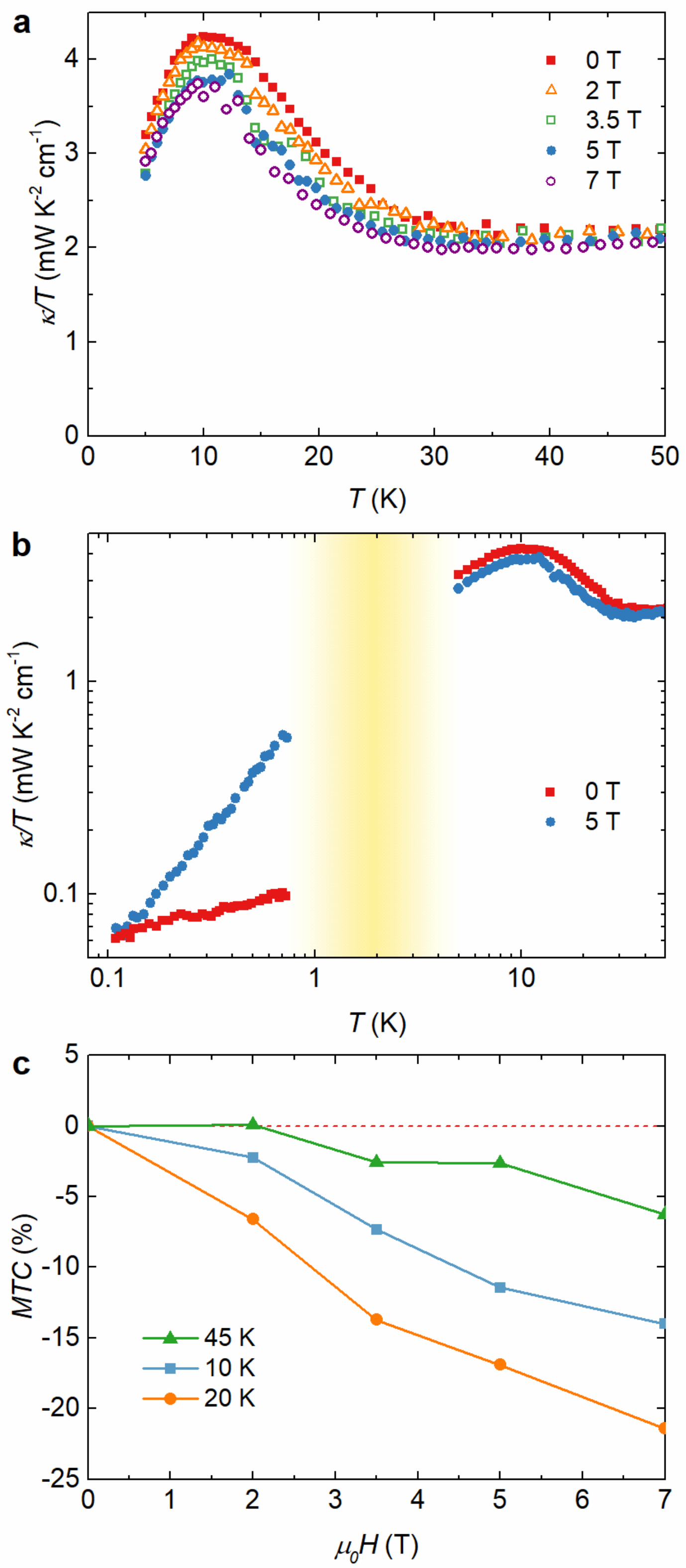

Supplementary Figure 5 | High-temperature thermal conductivities of Sample B. a The thermal conductivity of Sample B at various magnetic fields between 5 K and 50 K. **b** The logarithmical plot of the ultralow-temperature thermal conductivity and the high-temperature thermal conductivity at 0

T and 5 T. The yellow part between 1 K and 5 K denotes the crossover region. **c** The magneto-thermal conductivity MTC = $(\kappa(H) - \kappa(0\mathrm{T}))/\kappa(0\mathrm{T}) \times 100$ % at various temperatures. Small negative MTCs are observed between 5 K and 50 K.
